# Notes and News

**Published:** 1899-12-30

**Authors:** 


### Notes and News.

(Collected and Communicated.)

Complaint is being made in the United States that the examination of the health of intending immigrants now made by medical officers abroad is a farce, and plans are being prepared at Washington for a more rigid examination being effected.

Nothing is sacred to the bacteriologist, and among the various by-paths of infection which he has recently rummaged out holy water now stands in the schedule. We have no doubt that Dr. Abba, of Turin, is perfectly correct in all that he says in regard to the microbes in the *bénitiers* which stand at the entrance to Roman Catholic churches, but we doubt whether, amid the multitudinous chances of infection which surround us on every side, the matter is of much importance. Still, there surely could be no harm in taking care that the water is changed fairly often, and that the vessel is made clean.

During the first five days of the new year St. Martin's Town Hall, Charing Cross, will be devoted to the purposes of the Livingstone Exhibition, where all sorts of objects bearing upon travelling in Africa, and the maintenance of health in tropical countries will be shown. The exhibition has been organised in connection with the Livingstone College, which for the past six years has given to missionaries going abroad a useful and practical course of instruction in the laws of health, and in such elements of medical treatment as are most likely to be useful to them when far removed from qualified medical help.

A letter from Sir Henry Burdett appears in the *British Medical Journal* of last week controverting the idea that the war funds have in any serious degree interfered with the income of the hospitals. He says, "I am glad to think and to say from my knowledge of the men that there are certainly not more than six hospitals, if so many, which would venture to urge within two months of the outbreak of war that they were receiving no contributions from the public owing to the war funds. Such an excuse condemns the source from which it originates. A well-administered voluntary hospital so organises its system of raising money that the money comes in throughout the whole year, with the exception perhaps of a few of the summer months."

The *Lancet* last week published with its obituary notice an admirable full-page portrait of the late Sir Richard Thorne Thorne.

The third Scandinavian Medical Congress will be held at Copenhagen in July, 1900. The congress will devote itself mainly to the subject of serumtherapy.

Australia is alarmed at the approach of the plague. The disease has broken out at Noumea in New Caledonia, but as this is 800 miles distant precautions should be as effective as they have been in countries where a far shorter distance separates them from infected spots.

Dr. Stephens and Dr. Christophers, who are appointed to serve on the Royal Society's Commission for the Investigation of Malaria, have departed for Sierra Leone. They have recently returned from Central Africa. The Liverpool investigators are also working in the same locality to which the Royal Society's Commissioners have now gone.

As a result of the Local Government Board inquiry into the circumstances of the outbreak of typhoid fever in Dublin, the Commissioners have stated their finding that the medical officer of health of the district failed in his duty to notify the epidemic at its commencement, and his resignation is required. They suggest that the sub-sanitary officer for the district and the inspector of dairies should also be desired to resign.

A preliminary meeting of those interested in tuberculosis has been held, with the Earl of Derby in the chair. As the result of the meeting a British Congress on Tuberculosis has been arranged to be held in the spring of 1901, the Prince of Wales having consented to act as president, and to open the congress in person. The co-operation of representative bodies interested in public health throughout the Empire will be invited, and steps are to be taken to obtain subscriptions. An organising council has been formed, of which the Earl of Derby is president, and Sir William Broadbent chairman.

Amid much that is disquieting in the news from the seat of war one point stands out brightly, namely, the satisfactory arrangements which have been made for the treatment of the wounded. On all hands it seems to be admitted that the utmost gallantry has been shown in removing the wounded from the field of battle, that when they have once got into the hands of the doctors they have received every possible attention, that dressing and other medical appliances have been plentiful, and that the surgical treatment has been most successful. The latter is a point in regard to which we have interesting evidence in the large nnmber of wounded who have already been able to take their places again among their fighting comrades. Nothing but careful attention to the details of antiseptic surgery could have produced such a result; and after all that has been said, somewhat hastily, as we think, in detraction of the army medical department, it must be very reassuring to the country at large to have this positive proof that things are being well done, and that surgical procedures are being properly conducted in the field hospitals as well as at the base.

